# An Ore Image Segmentation Method Based on RDU-Net Model

**DOI:** 10.3390/s20174979

**Published:** 2020-09-02

**Authors:** Dong Xiao, Xiwen Liu, Ba Tuan Le, Zhiwen Ji, Xiaoyu Sun

**Affiliations:** 1 Information Science and Engineering School, Northeastern University, Shenyang 110004, China; 1870623@stu.neu.edu.cn (X.L.); lebatuan1@duytan.edu.vn (B.T.L.); 20184150@stu.neu.edu.cn (Z.J.); 2 Liaoning Key Laboratory of Intelligent Diagnosis and Safety for Metallurgical Industry, Northeastern University, Shenyang 110819, China; 3 Institute of Research and Development, Duy Tan University, Da Nang 550000, Vietnam; 4 School of Resources and Civil Engineering, Northeastern University, Shenyang Liaoning 110000, China; sunxiaoyu@mail.neu.edu.cn

**Keywords:** ore image, conveyor belt, image segmentation, DUNet, residual connection

## Abstract

The ore fragment size on the conveyor belt of concentrators is not only the main index to verify the crushing process, but also affects the production efficiency, operation cost and even production safety of the mine. In order to get the size of ore fragments on the conveyor belt, the image segmentation method is a convenient and fast choice. However, due to the influence of dust, light and uneven color and texture, the traditional ore image segmentation methods are prone to oversegmentation and undersegmentation. In order to solve these problems, this paper proposes an ore image segmentation model called RDU-Net (R: residual connection; DU: DUNet), which combines the residual structure of convolutional neural network with DUNet model, greatly improving the accuracy of image segmentation. RDU-Net can adaptively adjust the receptive field according to the size and shape of different ore fragments, capture the ore edge of different shape and size, and realize the accurate segmentation of ore image. The experimental results show that compared with other U-Net and DUNet, the RDU-Net has significantly improved segmentation accuracy, and has better generalization ability, which can fully meet the requirements of ore fragment size detection in the concentrator.

## 1. Introduction

In the mining industry, the early production of minerals includes three steps: blasting, crushing and grinding. There are three crushing stages of ore with different fragment size requirements. All the material is transported by conveyor belts, which consumes a lot of energy. In addition, because the size of the feeding ports on the conveyor belt of some concentrators cannot be adjusted, large ore fragments can easily block the feeding port and damage the belt when ore enters the feeding port, which seriously affects the production efficiency and safety of the mine. In order to reduce energy consumption, improve production efficiency and ensure production safety, the concentrator needs a real-time and effective method to determine the ore fragment size and adjust the parameters of production equipment according to the fragment size. Compared to the ore pile, the ore on the conveyor belt is tiled on it, so the ore fragments are less blocked, which can more intuitively reflect the size of the fragments. Therefore, we choose the ore image of the conveyor belt as the research object.

Using machine vision methods to segment the ore image to obtain the size of each ore is an effective method. However, the production environment on the mine is complicated; the dust interference is large, the open-air equipment has a large impact on the light, the colors and textures of the ore are different, the boundaries of the ore fragments are blurred, etc., all of which pose major challenges to any ore image segmentation technique. In response to these problems, many image processing methods have been proposed: OTSU and its improvement method [1,2], cluster analysis [3], watershed and its improvement methods [4,5,6,7,8,9], and graph-based segmentation algorithms [10]. They can segment specific ore images, but they are limited and require precise parameter adjustments.

Deep convolutional networks have the characteristics of high efficiency and accurate results in image segmentation. FCN [11], U-Net [12], SegNet [13], deformable convolutional networks [14,15] and other semantic segmentation networks have been proposed one after another and can realize the end-to-end segmentation of images providing a great breakthrough in semantic segmentation. Xu et al. [16] used a U-Net deep convolutional network to solve the segmentation problem of ordinary stone images, realized accurate segmentation of rock images, and further verified the feasibility of deep learning in segmenting mineral images.

However, extensive research shows that U-Net networks have certain limitations, so scholars have proposed different U-Net model variants. Casella et al. [17] implemented an adversarial network consisting of two fully convolutional neural networks. One (segmenter) is a segmentation network inspired by U-Net and integrated with residual blocks. Miao et al. [18] proposed and evaluated an improved U-net model that includes a combination of squeeze and excite (SE) and ResNet blocks. Dash et al. [19] proposed an automatic psoriasis lesion segmentation method based on the improved U-Net architecture (PsLSNet). The architecture consists of a fully convolutional network with a depth of 29 layers. Sun et al. [20] designed a U-Net-based network architecture DRRNet, replacing simple skip connections with encoder adaptive blocks, and using densely connected fusion blocks in the decoder. Jeppesen et al. [21] proposed a U-net-based remote sensing network (RS-Net) for detecting clouds in optical satellite images. Liu et al. [22] proposed a liver CT sequence image segmentation algorithm GIU-Net, which combines an improved U-Net neural network model with graph cutting. The U-Net-based method proposed by Fang et al. [23] combines hybrid dilated convolution (HDC) and spectral normalization, which can use sharp structures and fine textures to fill missing areas of any shape. Hong et al. [24] used U-Net to develop a novel segmentation framework suitable for deep WMH. The segmented WMH was subdivided with ten-fold cross-validation, and its true accuracy rate is high. Ibtehaz et al. [25] replaced the sequence of two convolutional layers with MultiRes blocks and developed a novel architecture MultiResUNet. Liu et al. [26] proposed an improved osteoporosis diagnosis algorithm based on U-NET network. Fang et al. [27] developed an improved network architecture using a residual channel attention block (RCAB) based on the conventional U-Net method: residual channel attention U-Net (RCA-U-Net). Jin et al. [28] proposed DUNet for segmentation of retinal blood vessels under the influence of deformable convolutional neural networks.

Based on DUNet, in order to solve the problem of information loss between convolutions and protect the integrity of information, this paper introduces the residual structure of ResNet [29], and proposes a novel ore image segmentation model called RDU-net, which has better segmentation effect.

The structure of the rest of this article is as follows: in Section 2, we will introduce the production and preprocessing methods of the ore image dataset. Section 3 describes the network structure of RDU-Net and the method of ore image segmentation. It also briefly introduces the DUNet. The experimental results are shown in Section 4. We evaluated different image segmentation methods. The conclusions are given in Section 5.

## 2. Preprocessing and Production of Data Sets

### 2.1. Image Preprocessing

Deep neural networks can effectively learn from unprocessed image data, but if the image data is properly preprocessed, it will be more efficient. The image data set is the video data of the ore on the conveyor belt collected in the Taiyuan Iron and Steel Group’s open-air beneficiation plant, and the video data is taken as frames.

**Figure 1:**
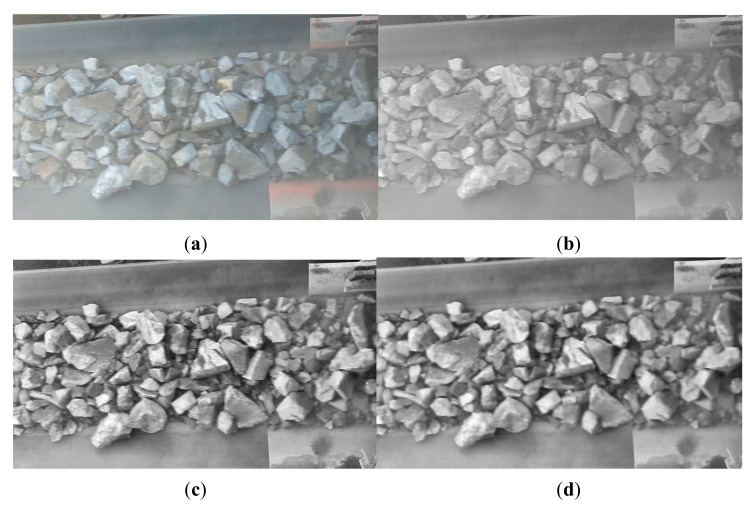
Images after each preprocessing step: (**a**) Original image, (**b**) Grayscale image, (**c**) Image after CLAHE operation, (**d**) Image after bilateral filtering.

It can be seen from Figure 1a that the ore fragments are uneven in color, the surface texture is complicated, the dust interference is serious, and the fragment edges are not obvious. These factors will affect the final segmentation effect. 

We use three image preprocessing methods. If the algorithm is directly processed with color images, the data processing time will be prolonged due to the large amount of data. A gray image requires only one byte to represent the gray value of a pixel, and also enhances the contrast with the background. Compared with RGB images, the amount of data to be processed is reduced by about two-thirds, which speeds up the algorithm operation. The processing result is shown in Figure 1b. We use contrast limited adaptive histogram equalization (CLAHE) to enhance the foreground-background contrast, remove dust interference, increase the contrast between various parts of images, enhance the image details in dark areas, and make the gap between ore fragments more visible as shown in Figure 1c. Finally, because the color of the ore is uneven, the edges are not clear, and the images contains a lot of noise, we need to perform noise reduction processing on the images, in order to remove the noise interference in the images as much as possible and maintain the details of the original images. It can be seen from Figure 1d that the use of bilateral filtering denoising not only effectively reduces the image noise, but also the edge information of the ore fragments is well retained.

### 2.2. Production of Data Sets

**Figure 2:**
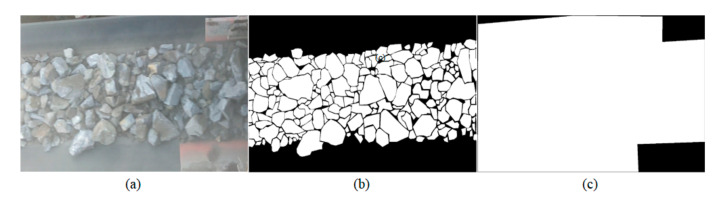
(**a**) Original images: (**b**) corresponding ground truth (**c**) field of view example.

In order to create a deep learning sample set and meet the needs of comparative analysis of experimental results, we take the belt section as the background and use the image labeling tool LabelMe to manually describe the boundaries of the ore fragments. At the same time, the mask is used to shield the area outside the image upload belt and keep the area within the field of view. Finally, the label maps and mask maps were obtained after binarization, as shown in Figure 2.

**Figure 3:**
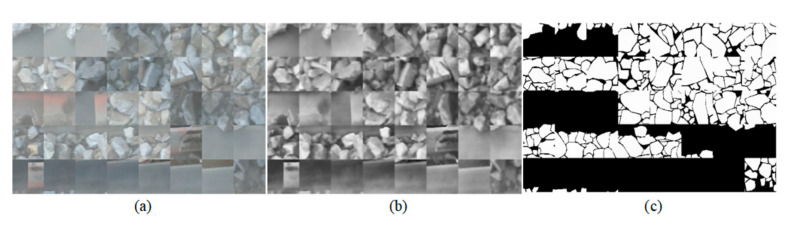
Typical 48 × 48 patches selected for model training: (**a**) shows the patches from the original images; (**b**) shows the patches from the preprocessed image; (**c**) shows the patches from the corresponding ground truth.

For the purpose of reducing the computational complexity and efficiently use the training data set while ensuring the surrounding local features, we set the size of the patches to 48 × 48 pixels, and intercepted 200,000 samples in total. All datasets are divided into a training set, a validation set, and a test set. 20 images in the dataset are used for training and validation and 10 images are used for testing. The training set is used to adjust the weights; the validation set is used to select the best weights; and the test set is used for performance evaluation. In order to reduce the problem of overfitting, RDU-Net is trained by randomly extracting small patches from images. It is shown in Figure 3.

## 3. The Establishment of RDU-Net

### 3.1. DUNet Model

**Figure 4:**
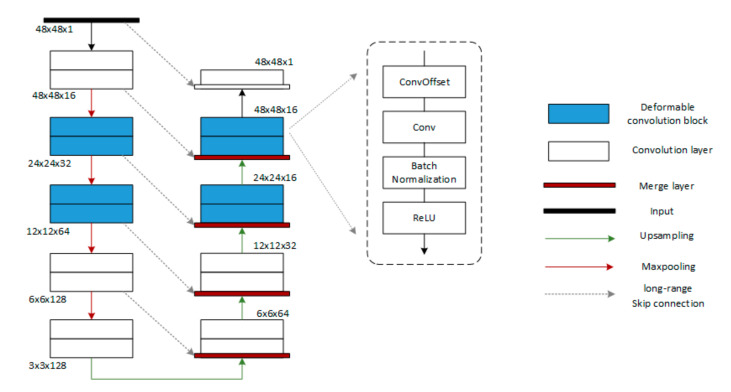
DUNet architecture with convolutional encoder and decoder based on U-Net architecture. Output size of feature map is listed beside each two layers.

The DUNet model is a deformed U-Net network model based on a fully convolutional neural network, which is applied to the segmentation of retinal blood vessels. Figure 4 illustrates the structure of DUNet. The architecture consists of a convolutional encoder (left) and a decoder (right) in the U-Net framework. At the bottom of the DUNet, a normal convolution layer is used instead of the deformation block. At each encoding and decoding stage, deformable convolutional blocks are used to simulate retinal vessels of different shapes and scales by learning local, dense and adaptive receiving fields. The dotted window shows the detailed design of the deformable convolution block. Each deformable convolution block includes a convolution offset layer (which is the core concept of deformable convolution), a convolution layer, a batch normalization layer [30], and an activation layer. With this structure, DUNet can learn to identify features and generate accurate retinal blood vessel segmentation results.

### 3.2. RDU-Net Model

In a convolution neural network, the deeper the network level is, the more errors will be generated in the training process, and the longer the training time is. ResNet, proposed by He et al. in 2015 [29], solves this problem to a certain extent. ResNet puts forward a method of fitting residual mapping, that is, it does not directly take convolution results as output, but uses residual mapping to perform calculation, which is called short cut. This residual connection solves the problem of performance degradation of deep convolution neural network under extreme depth conditions. 

This paper combines the characteristics of DUNet and residual connection, introduces the concept of “shortcut” into the DUNet structure, and proposes a novel model called RDU-Net. This model is based on the DUNet model, and the residual structure is added to the original variable convolution module (5–6, 8–9, 25–26, 27–28 layers). It helps the network to further extract features from the target, reduce the loss of information that occurs during the information transfer between the deep convolutional layers, and improve the accuracy of model detection. 

**Figure 5:**
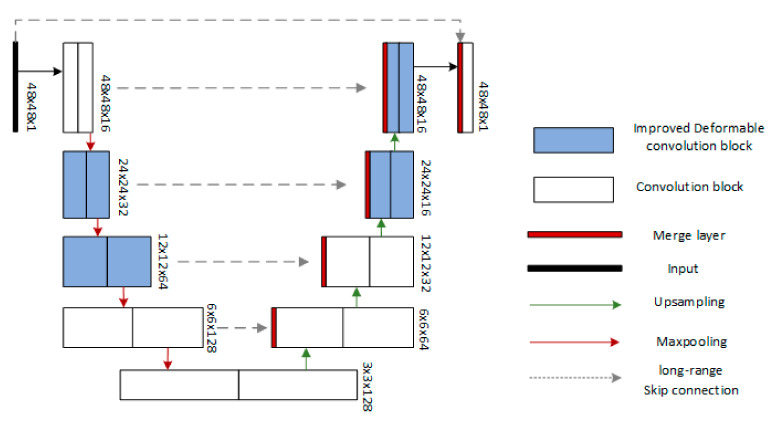
Overall structure of RDU-Net, Output size of feature map is listed beside each two layers.

This model uses a U-shaped structure, the left convolution module performs down-sampling to extract image features; the right convolution module performs up-sampling to improve the output resolution. We also use skip connection to combine low-level features with high-level features to extract contextual information to achieve pixel-level localization. Because deformable convolution is added to the model, the receiving field and sampling points can be adaptively trained to adapt to the size and shape of the ore fragments, both of which can achieve accurate segmentation. By using the residual structure, the shallow information extracted by the previous convolutional layer can be passed to the subsequent convolutional layer in a jumping manner, reducing the loss of information transmission between layers. Due to the characteristics of the residual network, the problem of performance degradation of deep convolutional neural networks is solved. This is also an important reason for choosing the residual structure to improve the DUNet model. Figure 5 illustrates the overall structure of RDU-Net.

**Figure 6:**
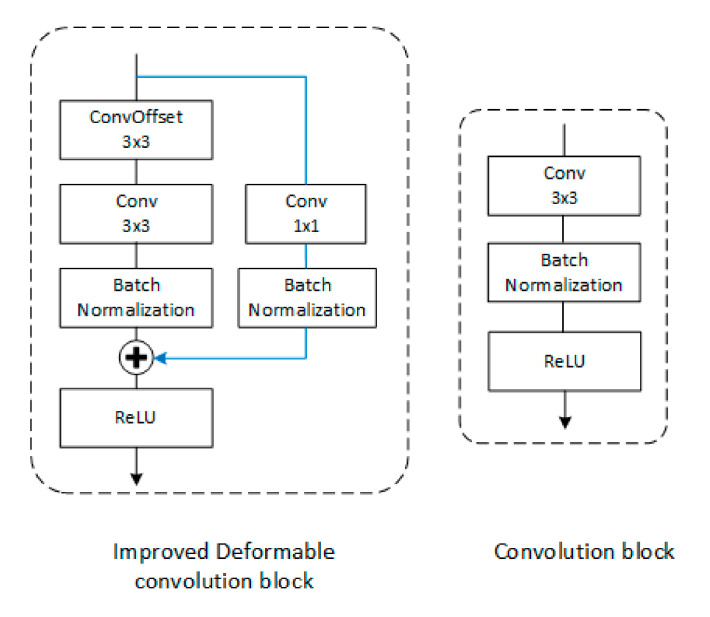
Detailed design of ordinary convolution block and improved deformable convolution block.

Figure 6 shows the detailed design of a normal convolution block and an improved deformable convolution block. Each improved deformable convolution block uses the convolution offset layer and the convolution layer to extract feature information, and uses residual connections to add the original feature map and the feature map after this structure, and simultaneously transfers shallow level information and deep level information to the next convolutional layer to extract features. Since the number of channels of the feature map after convolution becomes 2 or 1/2 of the original, this paper cannot use the method of ResNet to improve the network by adding several residual structures to a convolutional layer directly, but adds a 1 × 1 size convolutional layer to the residual connection, so that the dimension is unified when the residual is added to the current feature map. In order to solve the internal covariate shift problem and speed up the training processing speed, a batch normalization layer is inserted after each unit. The detailed structure of RDU-Net is shown in Table 1.

### 3.3. Algorithm Flowchart

**Figure 7:**
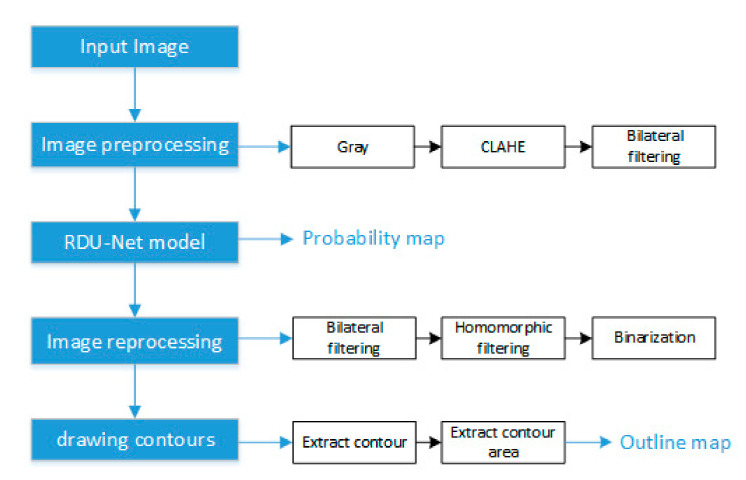
Algorithm flowchart.

The algorithm flow chart of this paper is shown in Figure 7. Firstly, the input image is preprocessed (gray, CLAHE, bilateral filtering) to make it easier to segment, and then it is transferred to the trained model to learn the recognition features and generate accurate segmentation results. Furthermore, in order to more fully evaluate the segmentation effect of the RDU-Net model, we need to accurately draw the outline of the ore fragments, so the probability maps output by the model need to be processed again (bilateral filtering, homomorphic filtering, binarization) to make the edges of the ore in the probability maps clearer. Finally, use OpenCV to find the outline of the ore fragments and draw it on the input image.

## 4. Experimental Results and Discussions

The computer used for image segmentation model training in this article is configured with an Intel Core i5-7500 3.40 GHz processor, NVIDIA GTX 1050 Ti graphics card, 16 GB RAM and a 500 GB Western Digital hard disk. In terms of software, the computer operating system is Windows 10 64 bit. The experiment is carried out under the pytorch framework. Opencv and PIL are used to process images. In terms of GPU, cudnn7.3.1 and cuda10.0 are used to accelerate the training and detection process. 

In the test process, the method of zeroing the boundary is used to make the length and width of the images an integer multiple of 48, so that the images of any resolution can be tested. The method of sliding window is used to intercept the test image samples, and the windows are partially overlapped, which makes the probability of the overlapped area points not only depend on the probability value of one test sample at this point, but also the average value of the probability of multiple samples containing the area points, so the results are more accurate.

### 4.1. Evaluation Criteria

Accuracy (ACC), positive predictive value (PPV), true positive rate (TPR), true negative rate (TNR) and the area under curve (AUC) of receiver operating characteristic (ROC) were used to evaluate the probability maps of model output.

ACC is a metric that measures the ratio between correctly classified pixels and total pixels in a data set. PPV, also known as precision, represents the proportion of true positive samples among all predictive positive samples. TPR, also known as sensitivity, measures the proportion of correctly identified locations, TNR, or specificity, measures the proportion of negatives that are correctly identified. These indicators are the accurate evaluation of the probability map of the model output in the numerical aspect. These indicators take the following form:

(1) $$ACC=\frac{TP+TN}{TP+FP+TN+FN}$$

(2) $$PPV=\frac{TP}{TP+FP}$$

(3) $$TPR=\frac{TP}{TP+FN}$$

(4) $$TNR=\frac{TN}{TN+FP}$$

Among them, TP indicates the number of true positive samples; TN indicates the number of true negative samples; FP indicates the number of false positive samples; FN indicates the number of false negative samples. In addition, the similarity and diversity of the test data set were evaluated by using F-measure (F1) and Jaccard similarity (JS).

In order to better quantify the segmentation effect, this paper counts the proportion of over-segmented and under-segmented ore fragments in the contour maps, which is called the error rate (Error). Among them, over-segmentation refers to that a piece of ore is divided into multiple blocks in the manual segmentation maps, and under-segmentation refers to that multiple pieces of ore are treated as one block in the prediction segmentation maps. Except for these two types, the ore fragments are segmented correctly, and the predicted segmentation boundaries are roughly the same as the artificial segmentation boundaries. We define CM as the number of correctly segmented ore fragments, US as the number of under-segmented ore fragments, and OS as the number of over-segmentation ore fragments. The calculation formula for the error rate is as follows:

(5) $$Error=\frac{US+OS}{US+OS+CM}$$

### 4.2. Evaluation of Model Segmentation Results

#### 4.2.1. Residual Structures are Located in Different Convolutional Layers

**Figure 8:**
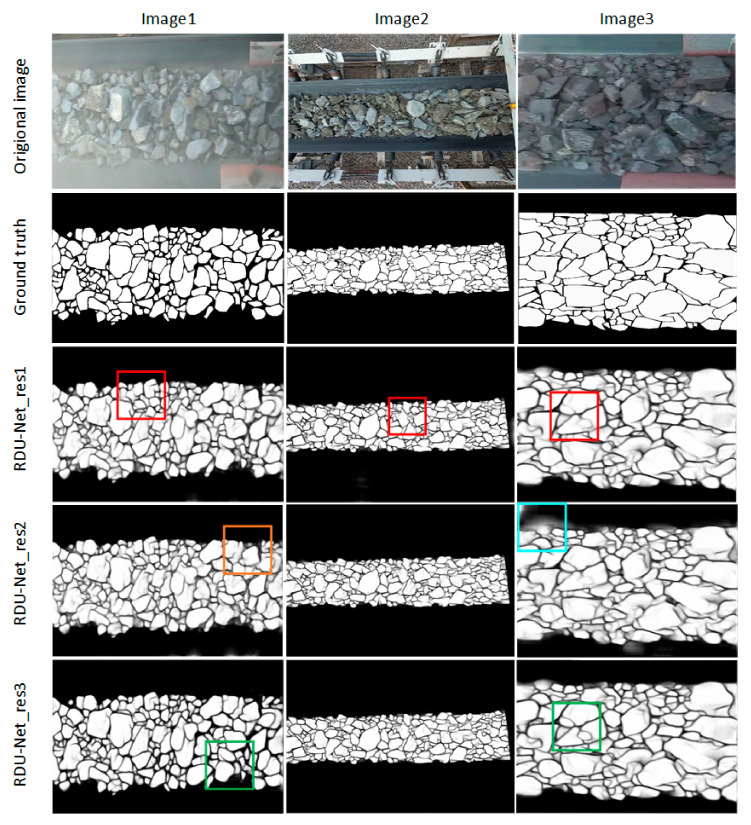
Comparison of model segmentation results with residual structure added at different positions. Red box: under segmentation; Orange Box: over segmentation; Blue box: wrong identification of conveyor belt; Green box: good segmentation.

In order to get the best segmentation effect. The position of the residual structure in the model is used as a variable, and many experiments are performed, divided into using the residual structure in all convolutional layers (RDU-Net_res1); using the residual structure only in ordinary convolutional layers (RDU-Net_2); using the residual structure only in deformable convolutional layers (RDU-Net_res3). The probability maps of the model output is shown in Figure 8. As can be seen from Figure 8 evidently, compared with other improved RDU-Net models, when the residual structure is only added to the deformable convolutional layer, the segmentation effect of the output probability maps is the best. The comparison of ACC, PPV, TPR, TNR, JS, FI and Test time of residual structure added in different positions is shown in Table 2. It can be seen from the table that the RDU-net network with residual structure only added in deformable convolution layers has reached the highest value in most indexes. The test times of each model are similar, among which, RDU-net_res3 is the fastest. In summary, we adopted the structure of RDU-net_res3.

#### 4.2.2. Comparison with Other Models

**Figure 9:**
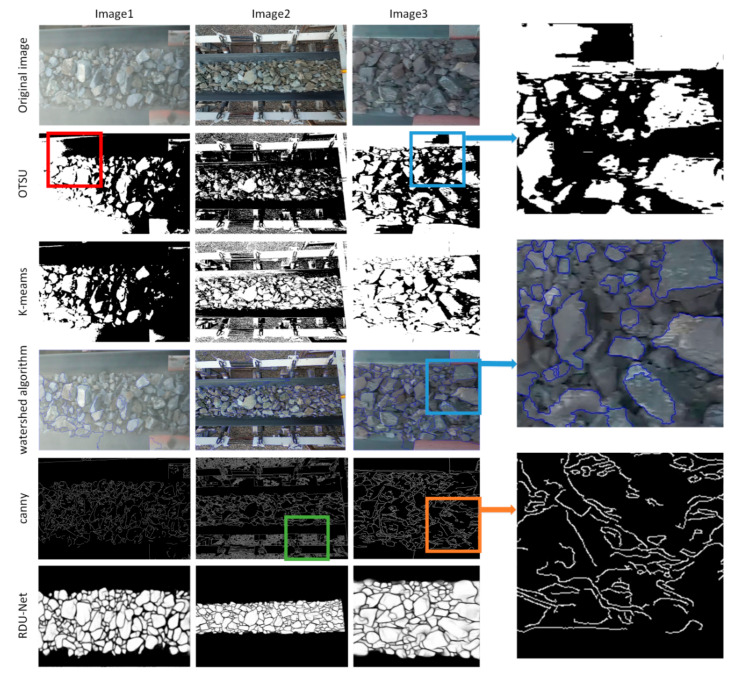
Comparison with Canny algorithm, Otsu algorithm, watershed algorithm and k-meams algorithm.

In order to explore the feasibility of the proposed RDU-Net ore image segmentation model, this paper makes a simple comparison with traditional segmentation methods such as canny contour extraction algorithm, OTSU, watershed algorithm, and K-means. As can be seen from Figure 9, Otsu, watershed algorithm and K-means algorithm can easily judge the dark part of the ore as the background, and the ore fragments are seriously adhered, and there are a lot of noise and under segmentation problems (Figure 9 blue box). Moreover, for images with uneven brightness such as image1, they cannot be well processed (Figure 9 red box). Canny edge detection algorithm will appear many discontinuous edges and will extract the wrong texture of the ore surface, but also cannot deal with the adhesive part (Figure 9 orange box). These algorithms cannot eliminate the influence of the conveyor belt and objects outside the conveyor belt (Figure 9 green box). The experimental results show that these traditional segmentation algorithms are not suitable for ore image segmentation on conveyor belt. The RDU-net algorithm proposed in this paper has a good segmentation effect for the ore image on the conveyor belt, which can effectively process the conglutinated ore fragments and effectively segment the ore fragments in the dark area, and perfectly shield the influence of the objects outside the conveyor belt and the conveyor belt.

**Figure 10:**
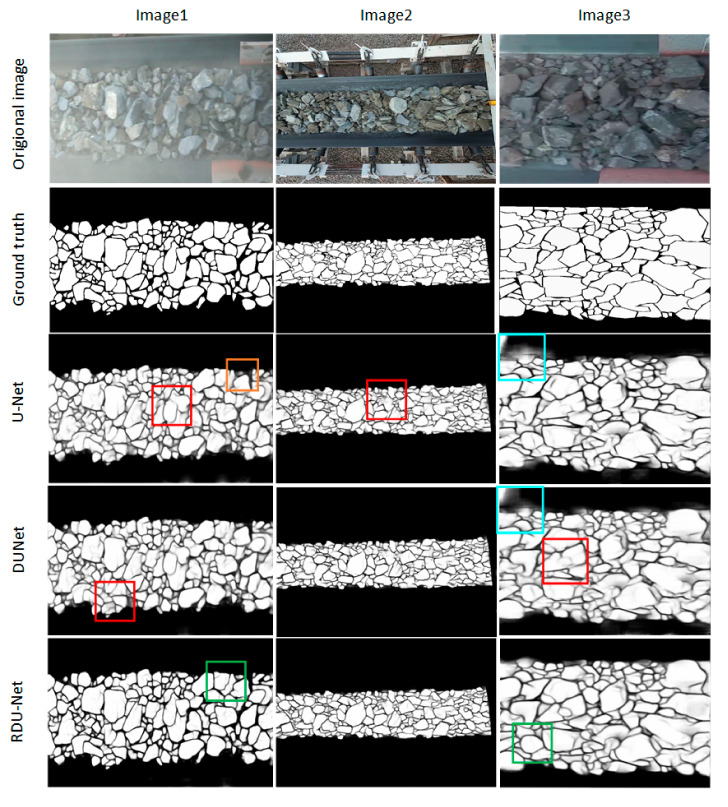
Comparison of segmentation results of U-Net, DUNet and RDUN-net. Red box: under segmentation; Orange Box: over segmentation; Blue box: wrong identification of conveyor belt; Green box: good segmentation.

This paper also compares the proposed RDU-Net model with U-Net and DUNet models. It can be seen from Figure 10 that the U-Net model has the worst segmentation effect, and there are many over-segmentations and under-segmentations. DUNet’s segmentation effect is better than U-Net, but both incorrectly identify some parts of the conveyor belt as ore. The RDU-Net model has the best segmentation effect, retaining more details, and the textures of the ore itself have not been recognized, which has better visual effects. Table 3 records the ACC, PPV, TPR, TNR, FI, JS, and Test time of the three models. As can be seen from the table, compared with the other two networks, Although RDU-Net is higher than the U-Net network in the test time, it has greatly improved the accuracy and is obviously better than the other two networks.

**Figure 11:**
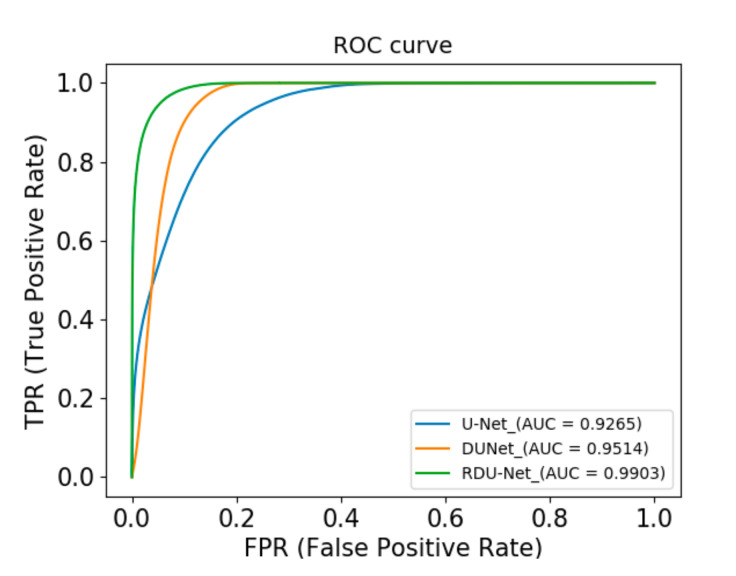
ROC curves of three models.

In addition, we use the ROC curve to evaluate the model. In ROC coordinates, the closer the ROC curve is to the upper left boundary, the more accurate the model is. As shown in Figure 11, the RDU-Net curve is the upper left corner of the three models, and the U-Net curve is the lowest one of the three models. In addition, the Figure 10 also shows that RDU-Net has the largest area under the ROC curve (AUC). Based on the above, RDU-Net has significant performance in solving the problem of ore image segmentation.

### 4.3. Evaluation of Contour Extraction Results

**Figure 12:**
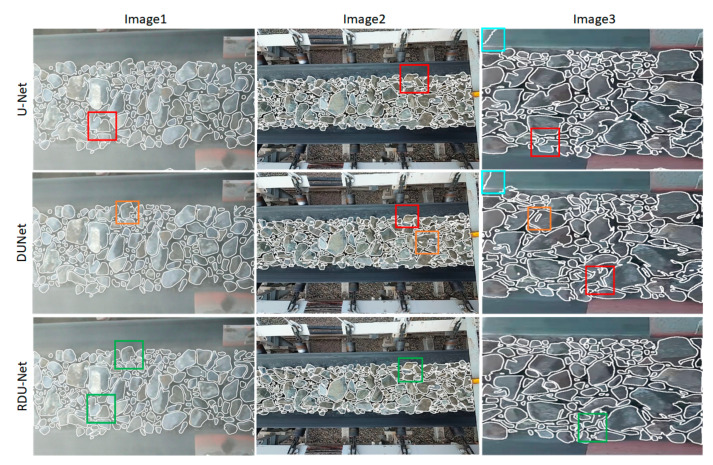
Comparison of contour maps divided by three models. Red box: under segmentation; Orange Box: over segmentation; Blue box: wrong identification of conveyor belt; Green box: good segmentation.

In order to better evaluate the RDU-Net model, the probability maps obtained after model training are processed by bilateral filtering, homomorphic filtering and binarization, and then uses OpenCV to find the area of all closed contours in the image and draw the ore contour maps. It can be seen from Figure 12 that the original U-net network cannot identify the edges of ore fragments well and cannot deal with the adherent part. There are many under-segmentation conditions, while the segmentation effect of DUNet network is better than that of U-net, but the over segmentation phenomenon is obvious, and some ore fragments with smaller size are not recognized. RDU-Net has a good segmentation effect for ore images, can effectively deal with stuck ore fragments, and can effectively segment ore fragments in dark areas. Although there is a tiny amount of under-segmentation and over-segmentation, It can more completely segment the ore contours on the conveyor belt, and the visual effect is good and satisfactory.

Table 4 summarizes the values of error, CM, OS, and US in the contour maps. The average segmentation error rate of RDU-Net is 2.90%, which is significantly lower than the other two models. These results show that the RDU-Net algorithm is accurate and feasible in the application of ore segmentation.

The RDU-Net segmentation algorithm is to help estimate the size distribution of the ore fragments. In this paper, the area of ore fragments in three test figures are counted and compared with the area of ground truth, and *p*-value is obtained, as shown in Table 5. It can be seen from the table that *p*-value > 0.05 indicates that there is no significant difference between the predicted value and the actual value, which meets the experimental requirements

**Figure 13:**
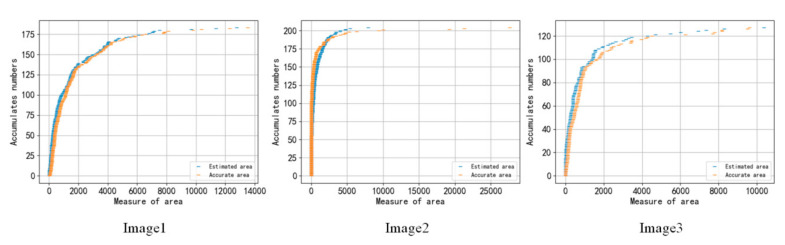
Cumulative size distribution of three test images.

In addition, the cumulative distribution of the area of the ore fragments obtained by the RDU-Net segmentation algorithm is calculated and compared with the artificial segmentation effect. As shown in Figure 13, the two curves are very close, which shows that the RDU-Net segmentation algorithm can play a good role in estimating the size of the ore fragments.

In addition, as shown in Figure 8, Figure 9, Figure 10 and Figure 12, we used two conveyor belt ore images with different styles from the training images for testing. After comparison with the artificial segmentation images, their error rates were 3.40% and 2.67%. Most of the ore fragments in the images are completely separated, and the results are satisfactory. This shows that the RDU-Net network has a strong generalization ability and can be used for the segmentation of ore images on different styles of conveyor belts.

## 5. Conclusions

This paper presents an ore image segmentation method based on the RDU-Net model. Firstly, the model preprocesses the training images to make it easier to process; secondly, it expands the size of data set by randomly extracting patches from the training image, and improves the accuracy of model segmentation by adding residual network structure on the basis of DUNet model; finally, the contours of ore fragments are extracted from the probability maps of model output to evaluate the segmentation results quantitatively.

Experimental results show that the RDU-Net network after adding the residual connection solves the problem of performance degradation of deep convolutional neural networks. Compared with DUNet and U-Net models, the accuracy of segmentation has been greatly improved. The RDU-net model provides an advanced method for the fragment size detection of ore. It is a powerful computer-aided system that can be used to study the crushing and clogging problems of concentrators. In the future, more ore image data will be merged to verify the proposed model, which is necessary to effectively reduce the calculation time. The next step will focus on how to reduce the detection time significantly while ensuring the accuracy of the detection, which will be a fruitful area for future work.

## 6. Patents

Published patent: a method for ore bulk rate detection based on RDU-Net model (Patent No. 201911096095.0).

## Figures and Tables

**Table 1:** Network structure of RDU-Net.

Contracting Path						Expanding Path					
Layer Number	Layer (Type)		Output Shape	Filter Size		Layer Number	Layer (Type)		Output Shape	Filter Size	
**L1**	**Input**		[1,48,48]			L16	Upsample-1		[128,6,6]	2 × 2	
L2	Conv-1+BN+ReLU		[16,48,48]	3 × 3		L17	Conv-15+BN+ReLU		[64,6,6]	3 × 3	
L3	Conv-2+BN+ReLU		[16,48,48]	3 × 3		L18	Conv-16+BN+ReLU		[64,6,6]	3 × 3	
L4	MaxPool-1		[16,24,24]	2 × 2		L19	Upsample-2		[64,12,12]	2 × 2	
L5	Conv-3+Conv-4+DeformConv-1+BN+ReLU	Conv7+BN	[32,24,24]	3 × 3	1 × 1	L20	Conv-17+BN+ReLU		[32,12,12]	3 × 3	
L6	Conv-5+Conv-6+DeformConv-2+BN+ReLU	Conv7+BN	[32,24,24]	3 × 3	1 × 1	L21	Conv-18+BN+ReLU		[32,12,12]	3 × 3	
L7	MaxPool-2		[32,12,12]	2 × 2		L22	Upsample-3		[32,24,24]	2 × 2	
L8	Conv-7+Conv-8+DeformConv-3+BN+ReLU	Conv7+BN	[64,12,12]	3 × 3	1 × 1	L23	Conv-19+Conv-20+DeformConv-5+BN+ReLU	Conv7+BN	[16,24,24]	3 × 3	1 × 1
L9	Conv-9+Conv-10+DeformConv-4+BN+ReLU	Conv7+BN	[64,12,12]	3 × 3	1 × 1	L24	Conv-21+Conv-22+DeformConv-6+BN+ReLU	Conv7+BN	[16,24,24]	3 × 3	1 × 1
L10	MaxPool-3		[64,6,6]	2 × 2		L25	Upsample-4		[16,48,48]	2 × 2	
L11	Conv-11+BN+ReLU		[128,6,6]	3 × 3		L26	Conv-23+Conv-24+DeformConv-7+BN+ReLU	Conv7+BN	[16,48,48]	3 × 3	1 × 1
L12	Conv-12+BN+ReLU		[128,6,6]	3 × 3		L27	Conv-24+Conv-25+DeformConv-8+BN+ReLU	Conv7+BN	[16,48,48]	3 × 3	1 × 1
L13	MaxPool-4		[128,3,3]	2 × 2		L28	Output = Conv-26		[1,48,48]	1 × 1	
L14	Conv-13+BN+ReLU		[128,3,3]	3 × 3							
L15	Conv-14+BN+ReLU		[128,3,3]	3 × 3							

**Table 2:** Performance of the three models tested.

	AUC	ACC	TNR	TPR	PPV	JS	FI	Test Time (s)
RDU-Net_res1	0.9848	0.9305	0.9372	0.9240	0.9392	0.9305	0.9315	20.66
RDU-Net_res2	0.9820	0.9244	0.9250	0.9238	0.9281	0.9244	0.9259	20.58
RDU-Net_res3	0.9902	0.9454	0.9623	0.9204	0.9430	0.9454	0.9316	20.16

**Table 3:** Performance of the three models tested.

	AUC	ACC	TNR	TPR	PPV	JS	FI	Test Time (s)
U-Net	0.9265	0.8229	0.8815	0.7706	0.8792	0.8229	0.8213	11.46
DUNet	0.9513	0.9012	0.8959	0.9103	0.8371	0.9012	0.8722	20.30
RDU-Net	0.9902	0.9454	0.9623	0.9204	0.9430	0.9454	0.9316	20.16

**Table 4:** Comparison of contour extraction results of test images.

Model	Image	CM	OS	US	Total	Error (%)
U-Net	Image1	161	7	19	187	13.90%
	Image2	196	10	17	223	12.10%
	Image3	93	5	7	105	11.42%
	Mean					12.47%
DUNet	Image1	177	13	2	192	7.81%
	Image2	199	16	7	222	10.36%
	Image3	101	6	3	110	8.18%
	Mean					8.78%
RDU-Net	Image1	183	3	2	188	2.65%
	Image2	227	5	3	235	3.40%
	Image3	109	1	2	112	2.67%
	Mean					2.90%

**Table 5:** *P*-value of three test images.

	Image1	Image2	Image3
*p*-value	0.26	0.34	0.39
